# Rho-of-plant activated root hair formation requires *Arabidopsis YIP4a/b* gene function

**DOI:** 10.1242/dev.168559

**Published:** 2019-03-11

**Authors:** Delphine Gendre, Anirban Baral, Xie Dang, Nicolas Esnay, Yohann Boutté, Thomas Stanislas, Thomas Vain, Stéphane Claverol, Anna Gustavsson, Deshu Lin, Markus Grebe, Rishikesh P. Bhalerao

**Affiliations:** 1 Umeå Plant Science Centre, Department of Forest Genetics and Plant Physiology, Swedish University of Agricultural Sciences, S-901 83 Umeå, Sweden; 2 Basic Forestry and Proteomics Research Center, Fujian Agriculture and Forestry University, Fuzhou 350002, China; 3 CNRS-University of Bordeaux, UMR 5200 Membrane Biogenesis Laboratory, INRA Bordeaux Aquitaine, Villenave d'Ornon 33140, France; 4 Institute of Biochemistry and Biology, Plant Physiology, University of Potsdam, Karl-Liebknecht-Str. 24-25, Building 20, D-14476 Potsdam-Golm, Germany; 5 Centre de Génomique Fonctionnelle, Plateforne Protéome, Université de Bordeaux, Bordeaux 33076, France; 6 Umeå Plant Science Centre, Department of Plant Physiology, Umeå University, SE-90 187 Umeå, Sweden

**Keywords:** ROP, YIP, Root hair, Secretion, Trans-Golgi network

## Abstract

Root hairs are protrusions from root epidermal cells with crucial roles in plant soil interactions. Although much is known about patterning, polarity and tip growth of root hairs, contributions of membrane trafficking to hair initiation remain poorly understood. Here, we demonstrate that the trans-Golgi network-localized YPT-INTERACTING PROTEIN 4a and YPT-INTERACTING PROTEIN 4b (YIP4a/b) contribute to activation and plasma membrane accumulation of Rho-of-plant (ROP) small GTPases during hair initiation, identifying YIP4a/b as central trafficking components in ROP-dependent root hair formation.

## Abstract (precis)

**Summary:** YPT-interacting proteins 4a and 4b (YIP4a/b) contribute to activation and plasma membrane accumulation of Rho-of-plant (ROP) small GTPases during hair initiation, identifying YIP4a/b as central trafficking components in ROP-dependent root hair formation.

## INTRODUCTION

Root hair formation in plants underlies strict spatial control and in *Arabidopsis thaliana* (Arabidopsis) root hairs emerge from the basal (root tip-oriented) ends of hair-forming epidermal cells. Members of the Rho-of-plant (ROP) small GTPase protein family provide early markers for the specific plasma membrane domain marking the incipient site of hair initiation (Molendijk et al., 2001; Jones et al., 2002). The mechanistic framework underlying polar ROP localization has been studied in the context of tip growth (Gu et al., 2003; Hwang et al., 2010; Chang et al., 2013; Huang et al., 2013) and polar positioning of the root hair initiation site (Fischer et al., 2006; Kiefer et al., 2015; Stanislas et al., 2015). Regulation of ROP cycling between a GTP-bound active and a GDP-bound inactive form sequestered in the cytosol represent a key factor. However, little is known about trafficking to and regulation of ROP accumulation at the root hair initiation site. Here, we report that the trans-Golgi network (TGN)-localized YPT-INTERACTING PROTEIN 4a and YPT-INTERACTING PROTEIN 4b (YIP4a/b) contribute to activation and plasma membrane accumulation of ROPs, identifying YIP4a/b as central trafficking components in ROP-dependent root hair initiation.

## RESULTS AND DISCUSSION

**Fig. 1.**
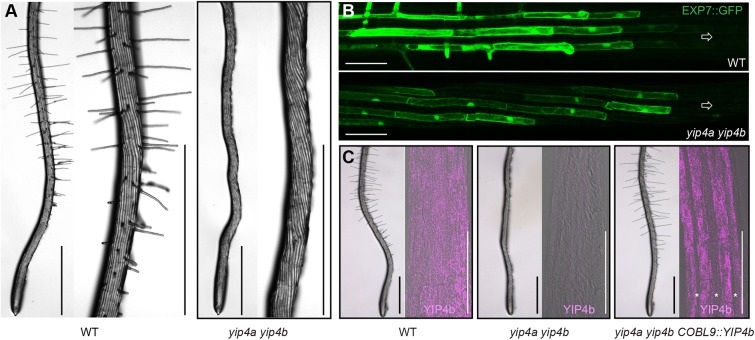
***YIP4a YIP4b* function in hair files is required for root hair development.** (A) Representative images of Col-0 wild-type (WT) and *yip4a yip4b* roots with higher magnification of the root hair zone (right panel). Scale bars: 1 mm. (B) Expression of *EXP7::GFP* in wild type and *yip4a yip4b*. Arrow indicates root tip orientation. Scale bars: 100 µm. (C) Roots of wild type, *yip4a yip4b* and *yip4a yip4b* expressing *YIP4b* under control of the *COBL9* promoter (*COBL9::YIP4b*). Image on the left shows root hair formation in the respective background. Scale bars: 1 mm. On the right, a confocal image (merged bright field and fluorescence in pink) of the root elongation zone (prior to hair formation) after immunolocalization with the YIP4b antibody is displayed. Scale bars: 100 µm. Note the lack of fluorescence in atrichoblast cells (marked with asterisks) in the *COBL9::YIP4b*-expressing line.

We have previously shown that the redundantly acting YIP4a and YIP4b proteins are required for cell elongation and act on secretory trafficking of some proteins and cell wall components via the TGN (Gendre et al., 2011, 2013). Strikingly, our analyses of *yip4a yip4b* double mutant roots indicated an almost complete absence of root hairs compared with wild type (WT) (Fig. 1A and Fig. S1A,B), with rare or no visible bulges, whereas the single *yip4a* and *yip4b* mutants have a similar or slightly higher hair density than wild type, respectively (Fig. S1A,B). Furthermore, expressing *YIP4a* under its own promoter is sufficient to restore hair formation (Fig. S1A,B). This suggests that both YIP4 proteins are required for hair initiation and act redundantly at an early stage.

In the *Arabidopsis* root, epidermal cell fate acquisition and subsequent differentiation into hair cells (trichoblast) or non-hair cells (atrichoblast) depends on their position relative to the underlying cortical cells. Mutations that result in a failure to specify trichoblast identity cause the formation of fewer or no hairs and ectopic hair cell specification results in additional hairs. Analyses of the expression pattern of a root hair file-specific marker, the promoter of *EXPANSIN7* driving green-fluorescent protein (*EXP7::GFP*) (Cho and Cosgrove, 2002; Singh et al., 2008) revealed that *EXP7::GFP* expression starts immediately prior to the formation of the first hair bulges and continues during tip growth, but is absent from non-hair cell files (Cho and Cosgrove, 2002) (Fig. 1B). The pattern of *EXP7::GFP* expression was not affected by loss of *YIP4a* and *YIP4b* function but, unlike in wild type, expression ceased once cells had fully elongated (Fig. S1C). Moreover, the expression of *YIP4b* driven by the trichoblast-specific *COBL9* promoter was observed in trichoblast cell files, as expected (Fig. 1C). These results indicate that it is unlikely that defects in root hair formation in *yip4a yip4b* are due to a failure of epidermal cell type specification.

Immunostaining employing a YIP4b antibody revealed ubiquitous YIP4b expression in elongating hair and non-hair cells prior to hair formation (Fig. 1C) compared with the absence of signal in the *yip4a yip4b* double mutant at the same differentiation stage. However, expression of YIP4a or YIP4b from the trichoblast-specific *COBL9* promoter in the *yip4a yip4b* background was sufficient to completely restore root hair development (Fig. 1C and Fig. S1A,B), suggesting that hair cell-specific expression of YIP4 is sufficient for YIP4 function in root hair development.

**Fig. 2.**
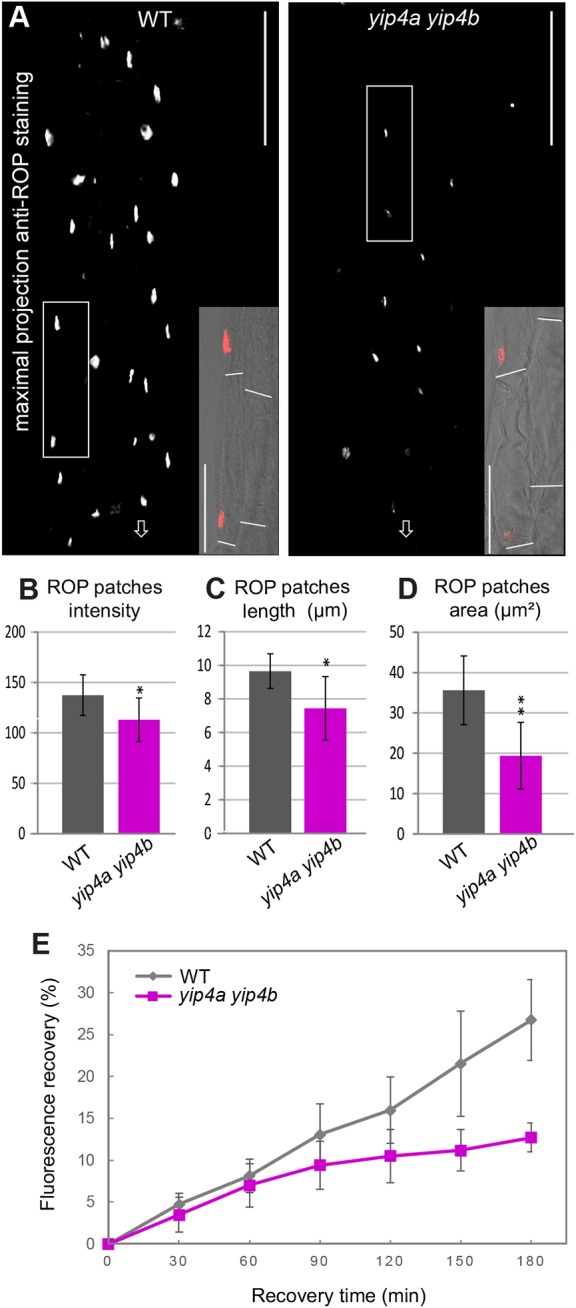
***YIP4a* and *YIP4b* mediate ROP accumulation in plasma membrane patches at hair initiation sites.** (A) Representative images of maximum projections (flattened image of the whole root depth) of a wild-type and a *yip4a yip4b* root immunolabelled using anti-ROP antibody. The region spanned 425 µm from the beginning of the elongation zone, prior to root hair emergence. Arrows indicate the root tip. Scale bars: 100 µm. Insets show a detail of one optical section of the root, the position of which is indicated by a corresponding white frame in the maximum projection with ROP immunolabel (red) merged with the bright-field image. Cell ends are visualized using a white line. Scale bars: 50 µm. (B-D) Measure of the intensity (B), the length (C) and the area (D) of the existing patches in wild-type Col-0 (WT) and *yip4a yip4b* from 30 roots (10 roots per biological replicate). Data are average±s.d. (*n*=30) analysed using the Kolmogorov–Smirnov test (**P<0.01, *P<0.05). (E) Quantification of fluorescence recovery after photobleaching in wild-type and *yip4a yip4b* roots expressing *ROP2::EYPF-ROP2* (*n*=5 roots with four cells for each root). Representative images can be seen in Fig. S5D.

Following hair cell specification, comes hair initiation marked by bulging at the site of root hair formation, tip-growth and growth cessation (Grierson et al., 2014). As the absence of visible bulges in *yip4a yip4b* indicated that YIP4s may act at an early stage of root hair development, we investigated the recruitment of ROPs to the basal end of trichoblasts, preceding the formation of the bulge (Molendijk et al., 2001; Jones et al., 2002). We employed an anti-ROP antibody (Kiefer et al., 2015) directed against a conserved epitope in ROP2, ROP4 and ROP6. In cells exiting the meristematic zone, ROPs concentrate into patches at the basal end of the cell before a hair bulge is visible and remain concentrated at the tip of the bulge and in the growing hair (Molendijk et al., 2001; Jones et al., 2002) (Fig. S2A,B). Along the first 900 µm of the root tip, the length chosen to cover hair initiation until the first bulges become visible, *yip4a yip4b* had 2.9 times fewer ROP patches than wild type (14.3±11.1 and 37.5±5.5, respectively; *n*=35 seedlings; *P*<0.001) despite an identical number of cells (Fig. S2C-E). Noticeably, although ROP patches start to be visible at the same distance from the quiescent centre in both *yip4a yip4b* and wild type (424±71 µm and 403±51 µm, respectively; *n*=4 biological replicates with seven seedlings each; *P*=0.5), they disappear after 811±208 µm in *yip4a yip4b* compared with 1065±161 µm in wild type (beginning of root hair bulging; *P*=0.1). Furthermore, the ROP patches present in *yip4a yip4b* were significantly weaker (Fig. 2A,B) and also smaller compared with wild type, as reflected by a reduced patch area and a reduced patch length (Fig. 2C,D). Thus, lack of YIP4 resulted in a reduction in the number of cells displaying ROP patches, a reduction of patch intensity and size, as well as a failure to maintain ROP patches as cells elongate but without affecting the polar basal placement of ROP patches along the trichoblast (*P*=0.986; Fig. 2A insets; Fig. S2F).

**Fig. 3.**
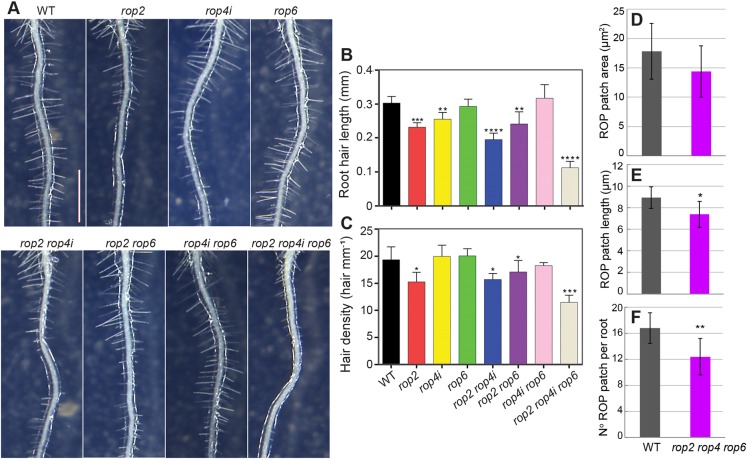
***ROP2*, *ROP4* and *ROP6* are required for root hair formation and elongation.** (A) Representative images of roots of wild type Col-0 (WT) and the following ROP mutants: *rop2*, *rop4i-3*, *rop6*, *rop2 rop4i-8*, *rop2 rop6*, *rop4i-5 rop6* and *rop2 rop4i-4 rop6*. Scale bar: 1 mm. (B) Measure of root hair length (in mm) in all genotypes mentioned above. More than 150 root hairs from at least ten growing roots for each genotype and each replicate (three replicates) were measured (*n*=450). (C) Hair density measurement (number of hairs per mm) for all genotypes mentioned above, *n*=30 roots per genotype (10 for each biological replicate). Data are mean±s.d. analysed using Student's *t*-test (*P<0.05, **P<0.01, ***P<0.001, ****P<0.0001). (D-F) Measure of the area (D), length (E) and number of existing patches (F) on a 480 µm region of wild-type and *rop2 rop4 rop6* roots, just after the meristem. Data are average±s.d. (*n*=30 roots for each genotype) analysed using Kolmogorov–Smirnov test (**P<0.01, *P<0.05). See Fig. S3B for representative images.

Testing the requirement of ROPs for root hair development has proved difficult, as ROP single and double loss-of-function mutants have no effect on root hair number (Kang et al., 2017). Among the 11 *Arabidopsis* ROP genes, *ROP2*, *ROP3*, *ROP4* and *ROP5* have been detected in root trichoblasts by transcriptomic analysis (Brady et al., 2007), and ROP2, ROP4 and ROP6 localize at the site of initiation even before bulges occur, when expressed as fluorescent protein fusions under the control of their own promoter (Xu and Scheres, 2005; Stanislas et al., 2015) or when detected by immunolabelling (Molendijk et al., 2001). Moreover, manipulating either their quantity, by overexpression, or the activity of ROP2, ROP4 and ROP6, by blocking them in a constitutively active (CA) or inactive (dominant negative, DN) state, has profound effects on root hair development. For example, both CA-ROP4 and CA-ROP6 expression induces root hair swelling (Molendijk et al., 2001) and DN-ROP2 expression results in fewer and shorter hairs, whereas CA-ROP2 plants produce more and longer hairs than wild type (Jones et al., 2002). To observe the effects of loss of function of ROPs, we examined root hair length and root hair density in *rop2*, *rop4* and *rop6* single, double and triple mutants (Ren et al., 2016) (Fig. 3A-C). Root hair length, which provides a measure of ROP action on tip growth, was reduced by 20% in *rop2* and *rop4i-3* (an RNAi line displaying downregulation of *ROP4*, Fig. S3A) when compared with wild type. Hair length was reduced by about 40% in the *rop2 rop4i-8* line and by about 70% in the *rop2 rop4i-4 rop6* triple line (Fig. 3B). Hair density, scored as the number of hairs per mm root, reflects the action of ROP on hair initiation and was decreased by 22% in *rop2* compared with wild type (Fig. 3C) but was not enhanced by additional downregulation of *ROP4* or *ROP6*. However, *rop2 rop4i-4 rop6* triple mutants displayed a 40% reduction in hair density compared with wild type. Thus, *ROP2*, *ROP4* and *ROP6* act redundantly on root hair length and root hair initiation. Interestingly, the redundancy in ROP signalling in root hair formation differs from that in leaf epidermal patterning, in which ROP2/4 and ROP6 act antagonistically (Fu et al., 2005), but is similar to that observed in petal development, in which they also act redundantly (Ren et al., 2016). *ROP4* expression varied between *rop4i* lines (Fig. S3A), rendering a direct comparison difficult. Nevertheless, *rop2* mutation had the strongest individual impact on hair density and length (Fig. 3A-C), while the overall strongest phenotype was observed in the *rop2 rop4i-4 rop6* triple mutant, strongly suggesting a role for *ROP4* in hair initiation and expansion (Fig. 3A-C). However, neither *ROP4* downregulation nor *rop6* loss of function individually affected the *rop2* mutant root hair phenotype (Fig. 3A-C).

The presence of root hairs in *rop2 rop4i-4 rop6* suggests that either additional ROPs are involved in root hair development or that residual ROP4 activity, owing to downregulation by RNAi, may account for the presence of the remaining hairs in the triple mutant. Indeed, size and number of the ROP patches were significantly decreased by 18 and 26%, respectively, in *rop2 rop4i-4 rop6* compared with wild type (Fig. 3D-F and Fig. S3B) but to a lesser extent than what was observed for *yip4a yip4b* (24 and 61%, respectively; Fig. 2C,D). These results indicate that the attenuation of ROP at the plasma membrane can significantly contribute to the lack of hairs in *yip4a yip4b*. Although ROP localization is not completely abolished, the *yip4a yip4b* mutant is almost hairless. Thus, additional YIP4-dependent factors may be required to proceed to tip growth. Alternatively, the quantity of ROP present in the patch is not sufficient or patches are not maintained long enough to trigger downstream processes in root hair development.

We next analysed the underlying cause for reduced ROP at the plasma membrane in *yip4a yip4b*. The amount of total ROP protein in wild-type and *yip4a yip4b* roots was not significantly altered (Fig. S4A,B). Importantly, the plasma membrane levels of EYFP-ROP2 were strongly reduced in *yip4a yip4b* compared with wild type (Fig. S5A-C), as observed for intrinsic ROP. Thus, reduction of ROP proteins at the plasma membrane in *yip4a yip4b* is not due to an overall reduction of ROP expression but is likely related to the secretory trafficking function of YIP4s. Whole-cell FRAP analyses bleaching the cell of interest and neighbouring cells expressing EYFP-ROP2 revealed that, after 180 min, fluorescence recovered to 26.8±4.9% in wild type compared with 12.7±1.7% in *yip4a yip4b* relative to the respective pre-bleach intensities. This substantial decrease in fluorescence recovery further supported the observation that plasma membrane delivery of newly synthesized ROP2 protein requires *YIP4a/b* function (Fig. 2E and Fig. S5D).

**Fig. 4.**
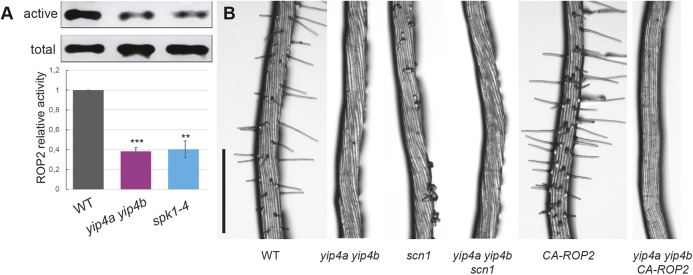
***YIP4a* and *YIP4b* contribute to ROP activation *in vitro* and to ROP activation of root hair development.** (A) Total ROP2 expression in roots of 5-day-old seedlings of wild type, *yip4a yip4b* and *spk1-4* (‘active’) compared with the active population of ROP pulled down by the effector RIC1 (‘total’). Five independent biological experiments were performed and quantifications are given in the graph below (*n*=5, data are average±s.d., ***P<0.001; **P<0.01, Student's *t*-test, two-tailed). Examples of full blots can be seen in Fig. S6. (B) Root hair phenotype in 5-day-old seedlings of Col-0 (WT), *yip4a yip4b*, *35S:CA-ROP2* and *scn1-1* mutants, and *yip4a yip4b CA-ROP2* and *yip4a yip4b scn1-1* triple mutants. Scale bar: 500 µm.

We then investigated whether the decrease of ROP at the plasma membrane could also be due to an attenuation of ROP activation in *yip4a yip4b*. ROP activity is positively regulated by guanine nucleotide exchange factors (ROP GEFs) that promote the exchange of GDP to GTP. Once active ROPs interact with effectors such as ROP-interactive CRIB-containing proteins (RICs), ROP inactivation relies on GTPase activation protein (ROP GAP) and inactivated ROPs are retrieved from the PM and sequestered in the cytosol by a guanine nucleotide dissociation inhibitor (ROP GDI) (Garcia-Mata et al., 2011). Constitutively activated ROP2 localizes to the PM in *Vicia faba* guard cells, whereas the dominant-negative variant is mostly cytoplasmic, indicating that the active form is predominantly membrane bound (Jeon et al., 2008). Moreover, reducing ROP activity by mutating *FERONIA* (*FER*), a receptor-like kinase interacting with ROP-GEFs, severely affected the number of hairs produced (Duan et al., 2010; Huang et al., 2013). Pull-down experiments in which only activated ROPs are pulled down, employing their effector RIC1 as a bait, revealed 60% less active ROP2 in *yip4a yip4b* roots compared with the wild type (Fig. 4A; Fig. S6), very similar to the level observed in the *spike1* mutant (*spk1-4*) defective in the ROP-GEF of ROP2 and ROP6 (Fig. 4A) (Ren et al., 2016). However, introducing the ROP-GDI mutant *supercentipede1* (*scn1-1*) or the constitutively active *CA-ROP2* form into the *yip4a yip4b* background did not restore hair growth. Instead, both the multiple bulge phenotype of *scn1-1* (Carol et al., 2005), as well as the multiple and ectopic hair phenotype of CA-ROP2, were suppressed in the *yip4a yip4b* background (Fig. 4B). Thus, genetic approaches that would enhance ROP levels by maintaining ROP at the plasma membrane via blocking its transfer to the cytosol (*scn1-1*) or by increasing the active pool of ROP (CA-ROP2) did not restore hair initiation. This, combined with a reduced level of active ROP in the *yip4a yip4b* mutant, strongly suggests that YIP4 function is required for ROP activity and ROP localization to the plasma membrane.

YIP4a and YIP4b localize to the TGN and mediate secretory trafficking from the TGN in *Arabidopsis* roots. Thus, the hairless phenotype of the *yip4a yip4b* mutant suggests that YIP4-mediated secretory trafficking plays an important role at an early stage of hair formation. Interestingly, proteomic analysis of a hairless barley mutant revealed that the SECRETION-ASSOCIATED AND RAS-RELATED PROTEIN 1A (SAR1A) GTP-binding protein and a vacuolar ATP synthase subunit B (V-ATPase) are crucial for hair initiation (Janiak et al., 2012). Homologs of these two proteins in *Arabidopsis* are involved in secretory trafficking and YIP4 colocalizes with V-ATPase at the TGN, supporting a role for TGN-mediated secretory trafficking in early hair development. Therefore, a reduced plasma membrane level of ROP or of its activator could be causing the root hair defects observed in *yip4a yip4b*.

To date, the mechanisms and factors underlying ROP localization at the plasma membrane remain unclear. For example, trafficking of ROP proteins from their site of synthesis on free polysomes in the cytosol to the plasma membrane was originally thought to be a direct process requiring post-translational lipid modification for membrane anchoring. The discovery that postprenylation modifying enzymes are intrinsic membrane proteins that are restricted to the endoplasmic reticulum (ER) called this view into question (Wright and Philips, 2006; Bracha-Drori et al., 2008). For animals, it has been proposed that Rho-type GTPases could reach the plasma membrane by two different means: at a slow rate through the vesicle trafficking machinery at the exit of the ER; and at a faster rate by a direct capture at the ER via GDI interaction (Garcia-Mata et al., 2011). In line with this, we detected ROP2/4/6 in SYP61-labelled secretory vesicles both by immunoprecipitation and by label-free quantitative proteomics (Fig. S7A-C). The low abundance of ROP2/4/6 in SYP61-labelled secretory vesicles when compared with YIP4a and YIP4b is consistent with the transient nature of ROP delivery through this compartment and the relatively small amount delivered to the plasma membrane. In contrast, YIP4s are resident proteins of the SYP61 compartment accumulating there at steady state. Our biochemical data together with the failure of *scn1-1* mutations or overexpression of ROP2-CA to increase root hair formation in the *yip4a yip4b* mutant strongly suggest a role for post-Golgi, TGN-mediated trafficking in ROP localization. Consistent with this hypothesis, interference with TGN function by locking the ARF1 GTPase either in the GTP- or in the GDP-bound form reduced fluorescence of EYFP-ROP2 at the plasma membrane (Xu and Scheres, 2005). The recent discovery that RAC5/ROP meets its effector at the TGN before being potentially exocytosed to the plasma membrane (Stephan et al., 2014) further supports the view that ROPs enter post-Golgi traffic, at least from the TGN. Furthermore, EYFP-ROP2 is trapped in brefeldin A (BFA) bodies enriched in TGN vesicles upon BFA treatment, suggesting that ROP trafficking involves the TGN (Xu and Scheres, 2005). However, TGN components that could mediate ROP localization at the plasma membrane have remained enigmatic. The root hair phenotype and the severe attenuation of ROP levels at the plasma membrane in the *yip4a yip4b* mutant provide evidence that post-Golgi trafficking via YIP4 is crucial for accumulation of activated ROPs in plasma membrane patches (Fig. S8 for schematic model). The functional importance of these finding is corroborated by the root hair density and length defects in mutants with reduced ROP2, ROP4 and ROP6 levels, highlighting the requirement of these ROPs for root hair development. Our work, thus, paves the way to further unravel the molecular interrelations between ROP activation and the vesicle trafficking machinery in plant development.

## MATERIALS AND METHODS

### Plant material

Plants were grown in soil (16/8 h light/dark; 24°C/19°C; 150 µE/m2/s; 70% humidity) or *in vitro* on 2.2 g/l Murashige and Skoog nutrient mix (Duchefa), 0.7% (w/v) plant agar (Duchefa), 0.5% (w/v) sucrose, buffered to pH 5.8 with MES (2-Morpholinoethanesulfonic acid, Sigma), after 2 days of vernalization. Seeds were surface sterilized in 70% ethanol, 0.1% (v/v) Tween 20 for 5 min and rinsed in 99% ethanol. The *Arabidopsis thaliana* Columbia-0 (Col-0) accession was employed as the wild-type background and mutants were genotyped according to the description in the respective articles: *yip4a-2 yip4b* (Gendre et al., 2013) (called simply *yip4a yip4b* in this article), *scn1-1* (Parker et al., 2000), *35S::CA-ROP2* (Li et al., 2001) and *rop2 rop4i-4 rop6* (Ren et al., 2016). The *rop4i-2*, *rop2 rop4i-8* and *rop4i-5 rop6* mutants were generated by independent *Agrobacterium tumefaciens*-mediated transformation using the *ROP4-RNAi* construct under the native promoter of *ROP4* (see Ren et al., 2016) introduced into Col-0, *rop2* (SALK_055328C) and *rop6* (SALK_091737C), respectively.

The following transgenic fluorescent-protein marker lines were used in the Col-0 background: *EXP7*::*GFP* (Cho and Cosgrove, 2002) and *ROP2*::*EYFP-ROP2* (Xu and Scheres, 2005).

### Root hair imaging and measurements

Five-day-old seedlings grown on half-strength MS agar plates were imaged using Leica MZ9.5 stereomicroscope coupled to Leica DC300 camera. For hair density of *rop* mutants, the number of hairs on the first 4 mm from the tip were manually counted on ten growing roots for each genotype in three independent biological replicates (*n*=30). For the *yip4* mutants and complementation lines, the number of hairs was counted from 5 to 10 mm away from the tip (*n*=20 seedlings from two biological replicates). Lengths from all hairs present on the first 4 mm from the tip were measured using ImageJ on ten growing roots for each genotype in three independent biological replicates with minimum 150 hairs measured per genotype (*n*=450). A Student's *t*-test was used to check for statistical difference (two-tailed with equal variance).

### Plasmid construction and plant transformation

1.9 kb of the *COBL9* promoter were amplified, as well as the *YIP4a* and *YIP4b* ORF using the following primers: COBL9pro-F-*Bam*HI, 5′-atggatcccaccaataatgtggcagatccgtagatct-3′; COBL9pro-R-*Asc*I, 5′-atggcgcgcctgtgtctttctccagagaaagttaag-3′; YIP4a-ATG-*Spe*I, 5′-atactagtatgtcacaaggcgatacag-3′; YIP4a-stop-*Not*I, 5′-atgcggccgctcaattgatggctatgatga-3′; YIP4b-ATG-*Spe*I, 5′-atactagtatgtcgcacaacgat-3′; and YIP4b-stop-*Not*I, atgcggccgctcaattaatggcaatgattaag.

Fragments were subsequently cloned into pGreen II 0229 (Basta resistance) by employing the following restriction sites: *Bam*HI-*Asc*I and *Spe*I-*Not*I, respectively. The *Agrobacterium tumefaciens* strain C58C1 was transformed with pGreen *COBL9::YIP4a* or *COBL9::YIP4b* and used to transform the *yip4a yip4b* double mutant using the floral dip method (Clough and Bent, 1998). The construct and plant transformation to get *yip4a yip4b* expressing *YIP4a::HA-YIP4a* have been described previously (Gendre et al., 2013).

### Confocal laser-scanning microscopy and immunolabelling

Fluorescence signals were viewed using a Zeiss LSM 780 confocal laser-scanning system mounted on a Zeiss Axio Observer Z1 inverted microscope, employing a water-corrected C-Apochromat 40× objective, numerical aperture 1.2 (Zeiss). GFP was detected using a 488 nm laser and 493-598 nm emission filter. *Arabidopsis* root whole-mount immunolabelling employed the protocol previously described (Gendre et al., 2011) with driselase treatment extended to 40 min for enhanced cell wall digestion in elongated root epidermal cells during ROP immunolocalization using rabbit anti-ROP at 1:250 (Kiefer et al., 2015), rabbit anti-YIP4b at 1:150 (Gendre et al., 2013) and anti-rabbit-DY633 at 1:100 dilution (Agrisera). DY633 was excited using a 633 nm laser and detected using a 638-759 nm emission filter.

### FRAP analysis

Wild-type and *yip4a yip4b* plants expressing *ROP2::EYFP-ROP2* (Xu and Scheres, 2005) were grown vertically on ½ MS agar plates. Five-day-old seedlings were mounted on a slide and overlaid with a 2 mm layer of MS agar to avoid dehydration. The seedlings were imaged with a Zeiss LSM780 confocal microscope using a water 40× objective (C-Apochromat 40×/1.2 W Corr M27). The rectangular region of interests (ROI) function of the Zeiss LSM software was used to select the area for bleaching. A 10- or 11-cell long strip in the transition zone along the entire width of the root was bleached (10-15 iterations of full power of the 458, 488 and 514 nm laser lines). Bleaching was repeated at different *z* positions to also ensure complete bleaching of underlying cells. Pre-bleach and post-bleach images were acquired as a *z*-stack to ensure imaging identical planes. Post-bleach images were acquired at 30 min intervals over 180 min. Recovery of plasma membrane intensity was calculated as a percentage of the mean intensity measured per individual cell relative to its pre-bleach intensity. A total of 20 cells from five individual plants (*n*=5) per genotype were quantified. The quantified cells had at least two fully bleached cells on either side to rule out recovery from lateral diffusion, etc. in neighbouring cells. Additionally, plasma membrane intensity of two non-bleached adjacent cells per root was determined and used to correct for loss of fluorescence caused by laser excitation during post-bleach image acquisition. Post-bleach intensity values were adjusted to set the intensity immediately after bleaching to zero.

### Pull-down assays

The ROP pull-down assays were performed as previously described (Ren et al., 2016). For quantification, data were analysed from five biological replicates using ImageJ software with the function of gel intensity analysis. The data are presented as the mean±s.d. (*n*=5) of the relative active ROP2 to the total amount of ROP. Statistical analyses were carried out using Student's *t*-test.

### Scoring of ROP patches

After immunolabelling, ROP patches along the first 900 µm region from the quiescent centre (QC), the distance of the first and of the last ROP patch to the QC, as well as the distance of the first root hair from the QC were manually measured with a Zeiss LSM780 using the position setup within the Zen software. The number of ROP patches was manually counted within the 900 µm distance throughout the depth of the entire root tip. As the data were not normally distributed, the significance of differences between distributions was tested using the non-parametric, two-sample Kolmogorov–Smirnov (KS)-test (www.physics.csbsju.edu/stats/KS-test.n.plot_form.html) on 35 seedlings originating from five biological replicates (seven roots per replicate). Positions of the ROP patches between the closest to and the most distant from the QC followed a normal distribution. Thus, statistical analysis was performed using a Student's *t*-test (two-tailed; sample with equal variance) on the average of the five biological replicates (*n*=5).

### Image analysis of ROP patches

ImageJ (imagej.nih.gov/ij/) was used to characterize the shape of each patch (area and length) and its associated fluorescence intensity. Maximal projections of confocal sections (*z*=35) from two consecutive tiles of 212 µm were each processed equally using a median filter after 8-bit transformation. Particles were isolated using a fluorescence intensity-based threshold and a size exclusion filter to remove all particles that were not considered as a patch but as intracellular punctae or noise. For each patch, surface area and average fluorescence intensity were measured and their maximal length was characterized using the Feret's diameter. The average and s.d. were calculated based on the pooled samples obtained from three independent replicates with 10 roots each (*n*=30). All ROP patches present in the root part analysed were measured and the average of all patches per root represents one sample (*n*). Significance was tested using the non-parametric, two-sample Kolmogorov–Smirnov (KS)-test (see above).

### Quantification of polar ROP patch position

Quantification of polar ROP patch position was performed as previously described (Kiefer et al., 2015). In brief, the distance between the basal end of the trichoblast and the basal end of the ROP patch was divided by the total trichoblast length. Measurements were performed on 90 cells (*n*=90) pooled and obtained from three experiments, each employing 10 roots from which three cells with clearly distinguishable cell boundaries and ROP patches were measured. Significance of differences between distributions was tested using a non-parametric, two-sample Kolmogorov–Smirnov (KS)-test (*P*<0.05).

### Western blot analyses

Western blots from gels loaded with 40 μg total protein extract per lane quantified by Bradford assay from roots of 7-day-old seedlings were incubated with rabbit anti-ROP (Stanislas et al., 2015) at 1:200 dilution followed by rabbit IgG horseradish peroxidase-linked whole Ab from donkey (GE Healthcare/Sigma-Aldrich, NA934) at 1:10,000 dilution. Blots were washed and detected with mouse monoclonal anti-α-tubulin antibody clone B5-1-2 (Sigma-Aldrich, T5168) at 1:3500 dilution and horseradish peroxidase-coupled AffiniPure goat anti-mouse IgG (Jackson ImmunoResearch Europe, 115-035-003) at 1:10,000 dilution. Enhanced chemiluminescence (ECL) prime western blotting detection reagents (Amersham) were used according to the manufacturer's instructions. Details on blot quantification can be found in the supplementary Materials and Methods.

### Quantitative PCR

Details on DNAse-treated total RNA extraction with an OMEGA Total RNA kit from five-day-old seedlings from three independent experiments, subsequent cDNA synthesis and quantitative PCR performed with a TaKaRa SYBR kit, as well as primer sequence information, can be found in the supplementary Materials and Methods.

### ROP detection in SYP61-positive immuno-purified vesicles

The immuno-purification procedure for SYP61-positive vesicles, label-free proteomics and LC-MS/MS detection of ROP peptides are described in the supplementary Materials and Methods.

### Accession numbers

Sequence data used in this research can be found in the TAIR database under the following accession numbers: *YIP4a*, AT2G18840; *YIP4b*, AT4G30260; *ROP2*, AT1G20090; *ROP4*, AT1G75840; *ROP6*, AT4G35020.

## Supplementary Material

Supplementary information
