# Supplementary material for: Rho-of-plant activated root hair formation requires Arabidopsis YIP4a/b gene function
Source: Development. 2019 Mar 11;146(5):dev168559. doi: 10.1242/dev.168559 (PMC6432664; doi:10.1242/dev.168559)
Supplement: Supplementary information [file develop-146-168559-s1.pdf]

Rho-of-plant-activated root hair formation requires *Arabidopsis YIP4a/b* gene function

Delphine Gendre[1,‡], Anirban Baral[1,*], Xie Dang[2,*], Nicolas Esnay[3], Yohann Boutté[3], Thomas Stanislas[4,Ŧ], Thomas Vain[1,†], Stéphane Claverol[5], Anna Gustavsson[6], Deshu Lin[2], Markus Grebe[4,6,‡], Rishikesh P. Bhalerao[1¶]

Supplementary Materials and Methods

Western blot quantification. Western blots from gels loaded with 40 µg total protein extract per lane quantified by Bradford assay from roots of 7-day-old seedlings were incubated with rabbit anti-ROP (Stanislas et al., 2015) at 1:200 dilution followed by anti-rabbit HRP (GE healthcare) at 1:10000 dilution. The blot was rinsed and then detected with mouse anti-tubulin antibody (Sigma) at 1:3500 dilution and goat anti-mouse (Jackson Immunoresearch) at 1:10000 dilution. Enhanced chemiluminescence (ECL) prime western blotting detection reagents (Amersham) was used according to the manufacturer's instruction. Images of the blots were acquired using a LAS-3000 imaging system (Fujifilm) and bands were quantified with ImageJ according to the following recommendations http://lukemiller.org/index.php/2010/11/analyzing-gels-and-western-blots-with-image-j/. The quantification is based on the average ± s.d. of n=6 biological replicates. Statistical difference was assessed with a Student's t-test (two-tailed distribution, two samples of equal variance).

ROP4 Quantitative PCR. DNAse-treated total RNA was extracted according to the manufacturer's instruction (OMEGA Total RNA kit) from five-day-old seedlings grown on 1/2x MS agar medium. RNA integrity was verified on an agarose gel and by the RNA 260 nm/280 nm OD ratio, while presence of residual gDNA was verified by PCR. The experiment was repeated three times. Quantitative PCR performed with TaKaRa SYBR kit was done on subsequent cDNA using the following primers for *ROP4* and *ACTIN2* (AT3G18780): ROP4-QRT-F 5'-ACCATCCTGGTGCAGTGCCTAT-3', ROP4-QRT-R 5'-ACACGCAGCGGTTCTTGTTCTT-3', Actin2-QRT-F 5'-TCCCTCAGCACATTCCAGCAGAT-3', Actin2-QRT-R 5'-AACGATTCCTGGACCTGCCTCATC-3'. Relative expression of *ROP4* to the *ACT2* level was set as 1. A Student t-test was performed (n=3) to assess statistical differences with ***P<0.001 and **P<0.01.

The semi-quantitative PCR was realized once on one of the biological replicates described above. The primers were as follows: ROP4-RT-F 5'-TGAGTGCTTCGAGGTTTATA-3', ROP4-RT-R 5'-CAAGAACACGCAGCGGTTCTTG-3', Actin2-RT-F 5'-CCGCTATGTATGTCGCCATCCAAG-3', Actin2-RT-R 5'-CCAGCAGCTTCCATTCCCACAAA-3'.

Detection of ROP in SYP61-positive vesicles. SYP61 vesicles were isolated employing the immuno-purification procedure described previously (Wattelet-Boyer et al., 2016). In brief, *Arabidopsis thaliana* seedlings were grown in liquid culture for eight days and then ground in vesicle extraction buffer (HEPES 50 mM pH 7.5, 0.45 M sucrose, 5 mM $MgCl_2$, 1 mM DTT, 0.5% (w/v) PVP (Sigma), 1 mM PMSF). Intact pools of light vesicles were collected at the 33/8% interface of a 38/33/8% sucrose step gradient after overnight centrifugation at 150 000 *g* at 4°C. This membrane fraction was then resuspended in the resuspension buffer (50 mM HEPES pH 7.4, 0.25 M sucrose, 1.5 mM $MgCl_2$, 150 mM NaCl, 1 mM PMSF, protease inhibitor cocktail) and the total membrane fraction was used as input for immuno-precipitation (IP). IP was performed with magnetic Dynabeads coupled to protein A (Invitrogen) according to the manufacturer's instructions. For each IP, 75 µl of beads were incubated with 7 µl of rabbit anti-GFP antibodies (Invitrogen) for 1 h with shaking at 4°C, washed with PBS-Tween (137 nM NaCl, 2.7 nM KCl, 10 nM Na_2HPO_4, 1.8 nM KH_2PO_4, 0.02 % Tween-20), equilibrated in the resuspension buffer for 10 min on ice and incubated with 1 ml of purified total membrane extract for 1 h with shaking at 4°C. Several washes were performed with 1 ml of resuspension buffer for 5 min with shaking at 4°C. Step gradient-purified total membrane fractions (IP input) and bead-immuno-purified fractions (IP output) were loaded at equal quantity on an SDS-PAGE gel and subjected to western-blotting. Purified rabbit anti-ROP antibody at 1:200 dilution was used to detect ROPs as described, previously (Kiefer et al., 2015).

Label-free proteomics was used to identify ROP2, ROP4 and ROP6 protein peptides in the SYP61 IP fraction. In brief, IP samples were treated with 25 µl 1% (w/v) SDS for 30 min at 37°C, 0.3 µl DTT 2 M was then added with subsequent incubation for 30 min at 37°C, 2.3 µl iodoacetamide 1 M was added followed by 30 min incubation at 37°C and finally 7 µl 5x Laemmli loading buffer was added followed by incubation for 30 min at 37°C. Samples were loaded and subjected to SDS-PAGE electrophoresis, extracted from the gel and injected into an LC-MS/MS Q-Exactive with a gradient time of 120 min. Label-free quantitative data analysis was performed on raw LC-MS/MS data imported in Progenesis QI for Proteomics 2.0 (Nonlinear Dynamics Ltd). Data was processed by volume integration for 2-6 charge-state ions and calculation of protein abundance (sum of the volume of corresponding peptides). Quantitative data was considered for proteins quantified by a minimum of two peptides.

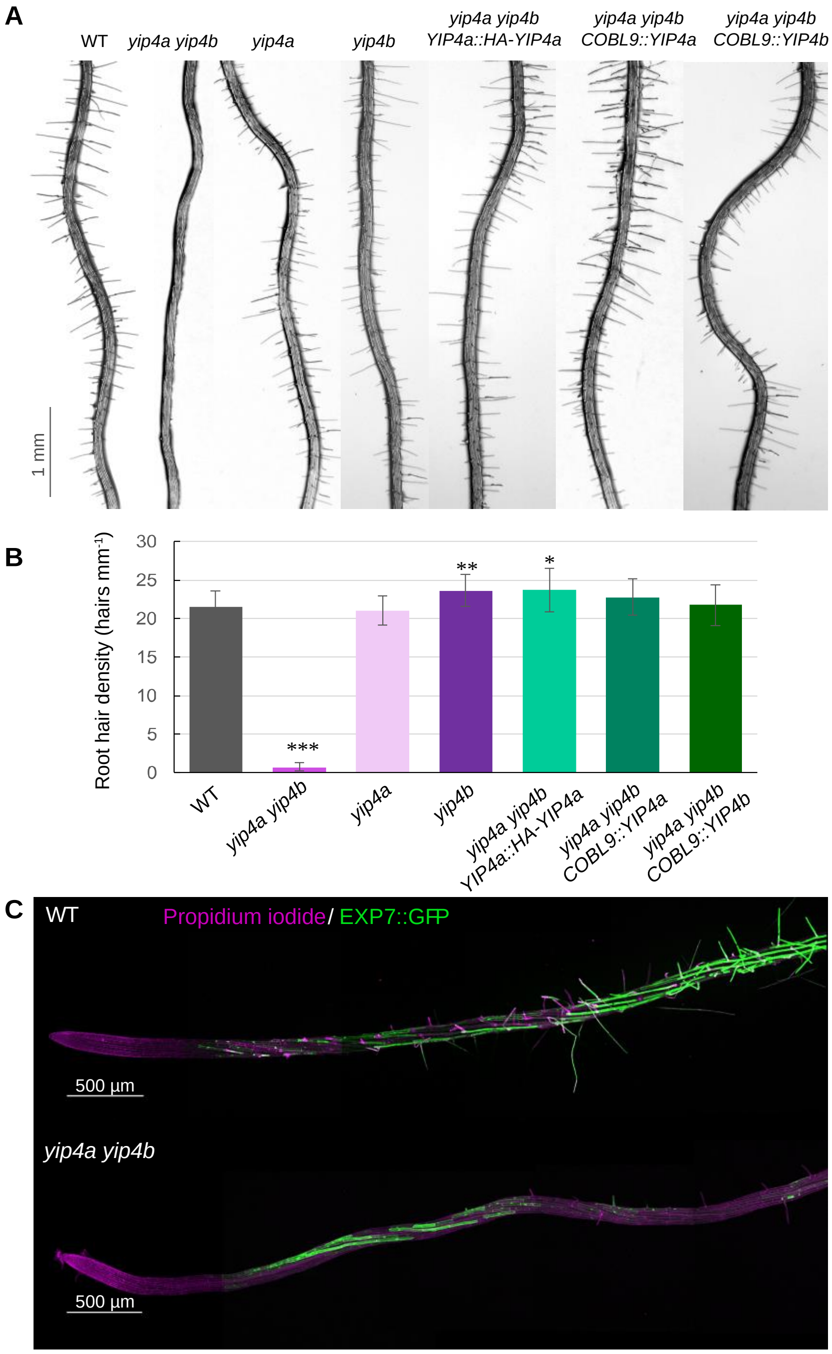

Fig. S1. Complementation of root hair density in *yip4a yip4b* expressing YIP4a and ceasing of *EXP7* promoter activity in differentiated *yip4a yip4* root hair cells.

(A-B) Representative images (A) and root hair density measurement (B) in WT, *yip4a yip4b, yip4a and yip4b* single mutants as well as *yip4a yip4b* double mutant complemented with *YIP4a::HA-YIP4a* and *COBL9::YIP4a/b*. Hairs were counted on a portion of 5 mm (from 5 to 10 mm away from the tip) on 20 plants per genotype (average ± s.d., *n*=20). Asterisks indicate statistical differences (****P*<0.001, ***P*<0.05, **P*<0.01 with Student t-test). (C) Representative images of WT and *yip4a yip4b* roots expressing *EXP7::GFP* (green). Propidium iodide counterstaining of cells walls (magenta).

Development • Supplementary information

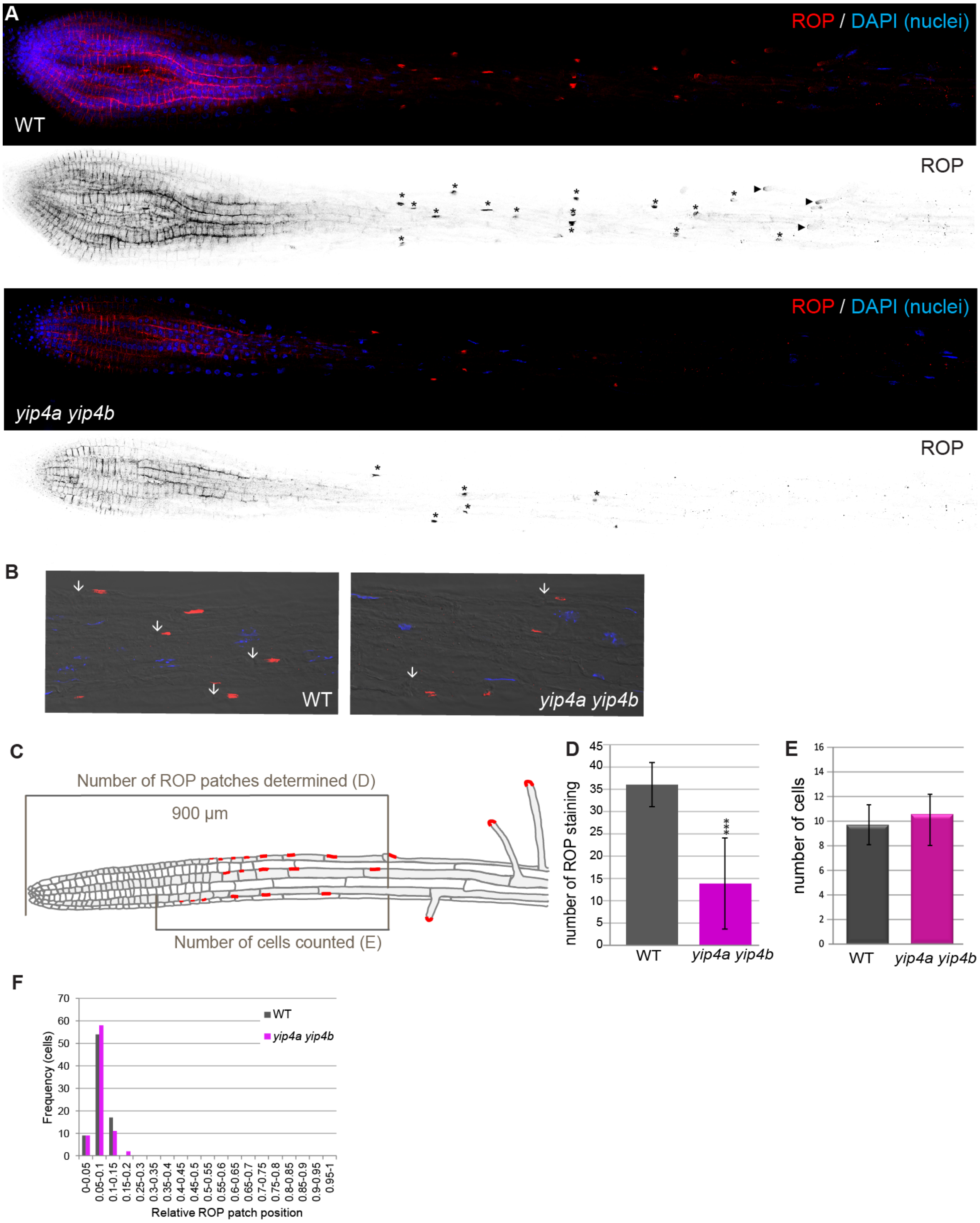

Fig. S2. Number of ROP patches in the wild type and in the *yip4a yip4b* mutant.

(A) Representative images of Col-0 (WT) and *yip4a yip4b* roots immunostained with anti-ROP antibody (red) and nuclei stained with DAPI (blue). Below the overlay of the anti-ROP and DAPI images, the inverted ROP greyscale signal corresponding to the image above with the patches marked with an

Development • Supplementary information

asterisk and the ROP signal at the tip of growing root hair is indicated with an arrow. (B) Close-up images with the transmission image merged to better visualize ROP patch positioning with the root tip pointing to the bottom. Arrows indicate basal ends of cells. (C) Scheme representing a typical WT root starting from the quiescent centre, indicating the region where the number of ROP (d) and the number of cells (e) were counted. (D) Average ± s.d. of the number of ROP patches counted on the first 900 µm of the root starting from the quiescent centre. n=35 seedlings (7 per biological replicate, 5 replicates). Kolmogorov-Smirnov test with ***P<0.001. (E) Number of post-mitotic cells in WT and *yip4a yip4b* along the first 900 µm of the root (n=15 roots, average ± s.d.). (F) Relative ROP patch positioning in WT and *yip4a yip4b* roots (n=90 cells; 3 cells per root, 10 roots in each of the three replicates). Non-significative difference P=0.986 with Kolmogorov-Smirnov test).

Development • Supplementary information

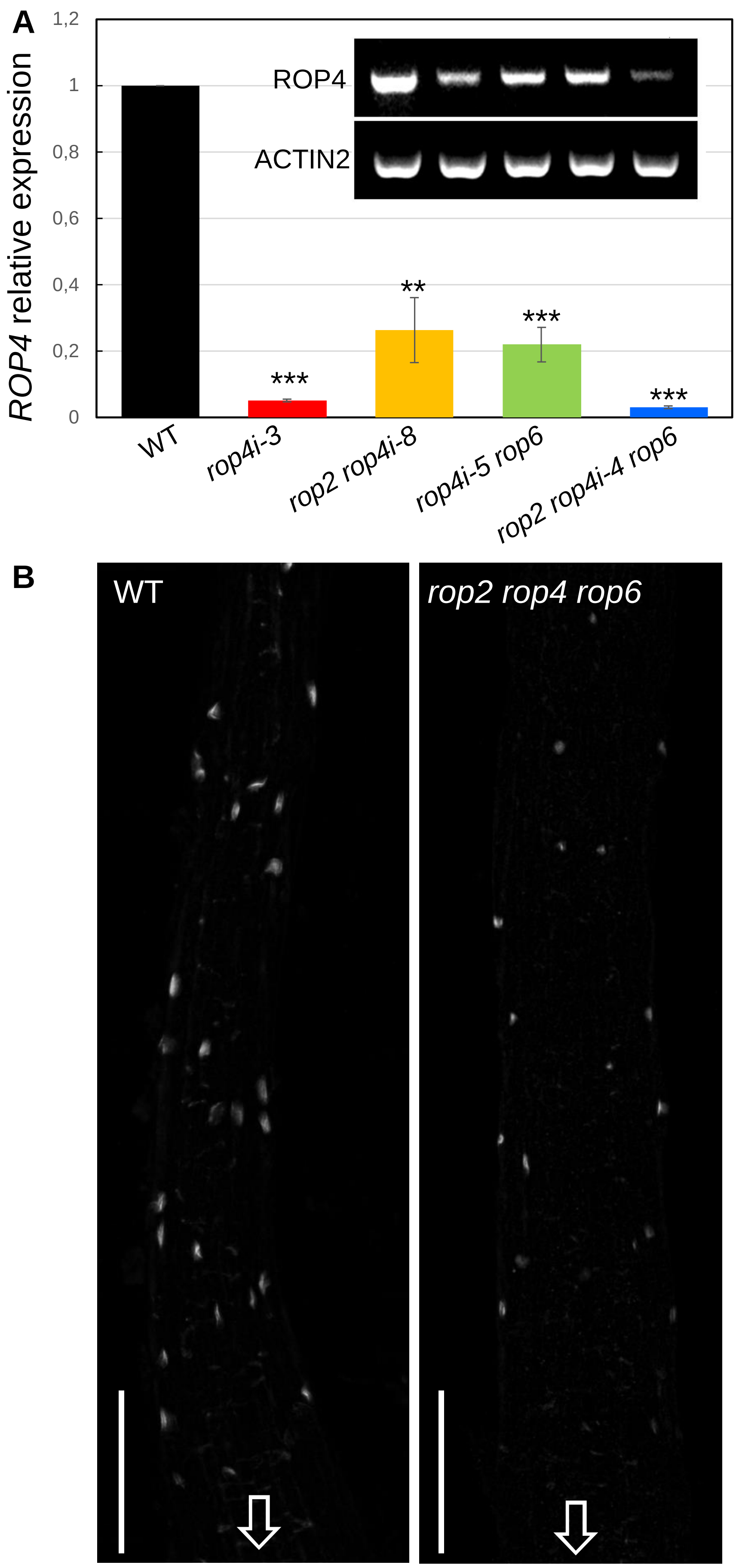

Fig. S3. ROP4 downregulation in *rop4i-3*, *rop2 rop4i-8* and *rop4i-5 rop6* and *rop2 rop4i-4 rop6*. (A) Quantitative real-time PCR analysis of the expression level of *ROP4* in Col-0 (WT) and the above-mentioned mutants. The expression level of *ACTIN2* is used as reference (n=3 biological replicates, ***P<0.001 with Student t-test). In inset, is a semi-quantitative PCR done on one of the biological replicates as visual reference. (B) Representative images of maximum projections (flattened image of the whole root depth) of WT and *rop2 rop4i rop6* roots immunolabeled with anti-ROP antibody. The region spanning 480 µm from the beginning of the elongation zone (prior to root hair emergence). Arrows indicate the direction towards the root tip. Scale bar, 100 µm.

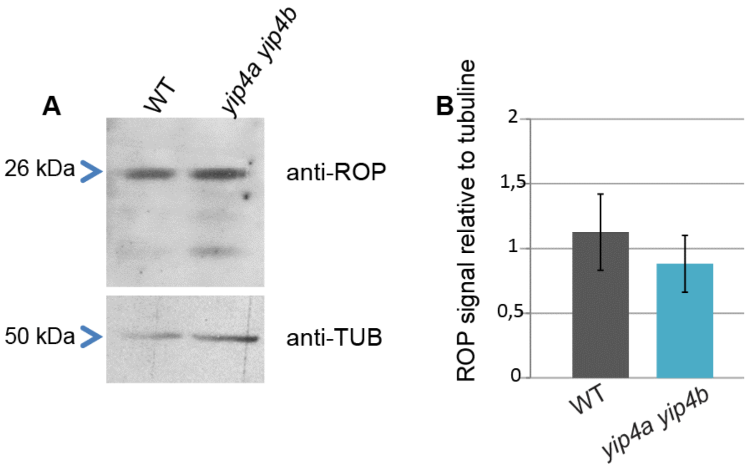

Fig. S4. The total amount of ROP remains unaffected in *yip4a yip4b*. (A) Representative Western blot showing anti-ROP and anti-tubulin (TUB) signals from roots of 7-day-old wild type (WT) and *yip4a yip4b* seedlings. (B) Quantification was performed with Image J based on 6 biological replicates (*n*=6, average ± s.d.) is displayed. A Student's t-test, two-tailed with equal variance, indicated no statistical difference (*P*=0.3).

Development • Supplementary information

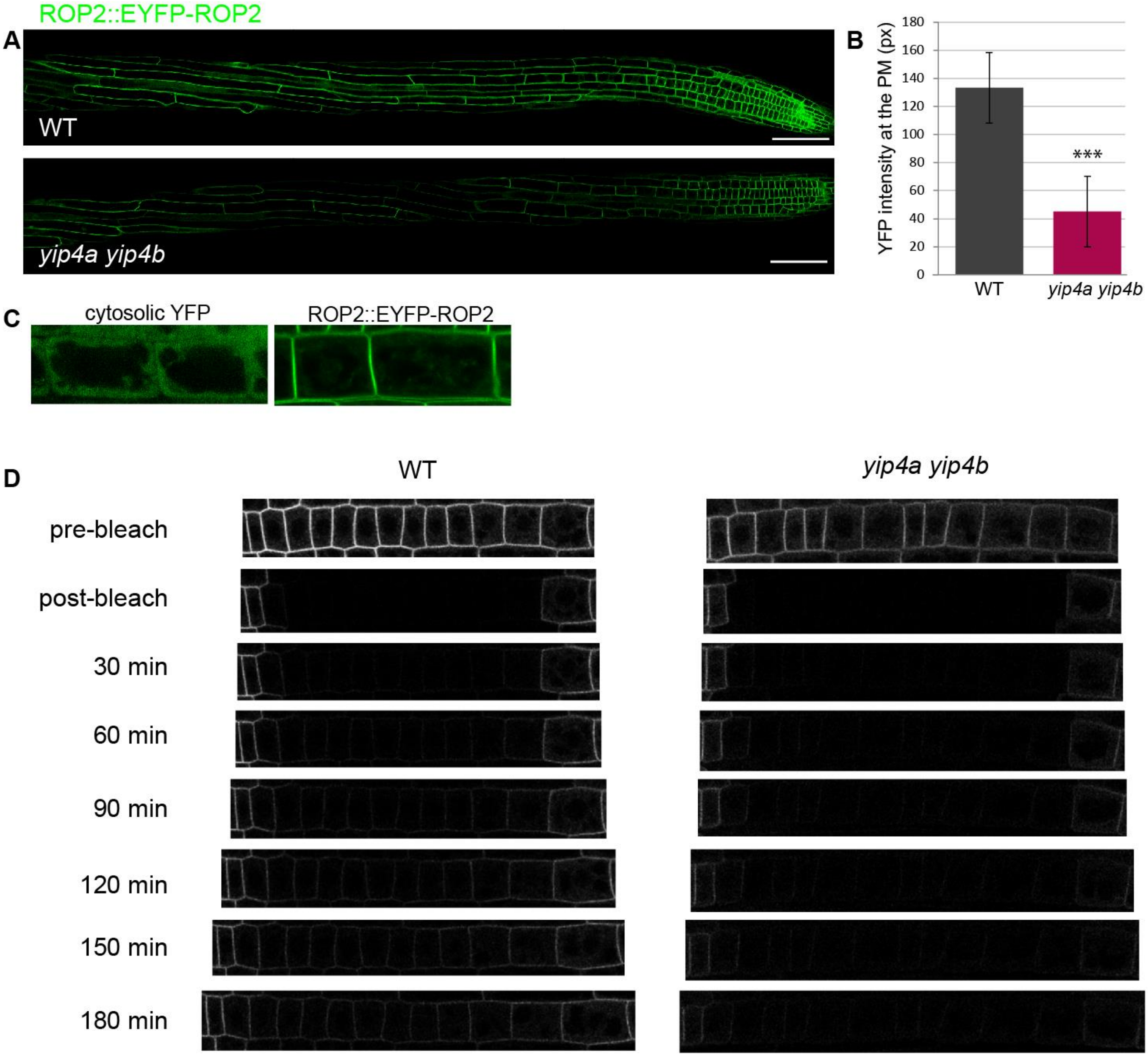

Fig. S5. *yip4a yip4b* mutants display less EYFP-ROP2 at the plasma membrane than wild type. (A) Representative image of *ROP2:EYFP-ROP2* line expressing EYFP-ROP2 in WT or in *yip4a yip4b*. Scale bar, 100 µm. (B) Fluorescence intensity quantification of EYFP-ROP2 in *ROP2:EYFP-ROP2* expressing lines at transversal plasma membrane around 400 µm from the root tip. n = 18 roots, 6 roots per replicate in 3 biological replicates. ****P*<0,001 calculated with Mann-Whitney U-test as the data were not normally distributed. (C) Distinction between cytosolic (cytosolic YFP) and EYFP-ROP2 PM signal in the cell-type used for quantification in (B), indicating that it is mostly PM signal that is quantified. (D) Representative images of a 10-11 cells long strip in the transition zone of WT and *yip4a yip4b* expressing EYFP-ROP2 before and after bleaching (the entire width of the root was bleached with 10-15 iterations at full power of the 458, 488 and 514 nm laser lines). Fluorescence recovery after 30, 60, 90, 120, 150 and 180 min is monitored. Note that the cells are growing during the course of the experiment, indicating that they are still alive and functional.

Development • Supplementary information

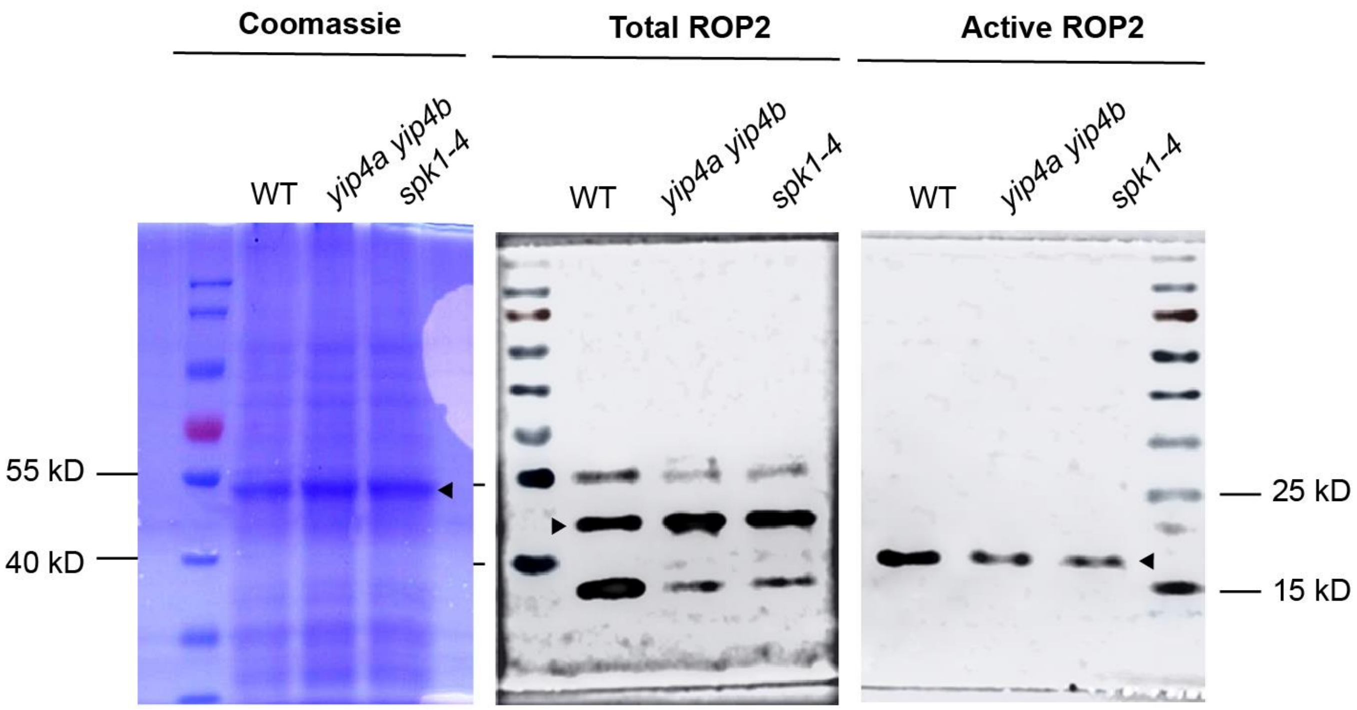

Fig. S6. Representative Western blots of the activated ROP2 pull down.

From left to right: Coomassie staining (arrowhead indicates Rubisco protein, 53 kDa), Western blot with ROP antibody on total protein extract (arrowhead indicates the band chosen for quantification in Fig. 4A), Western blot with ROP antibody on the RIC1-pulled fraction (arrowhead indicates the band chosen for quantification in Fig. 4A).

For ROP2 activity assays in WT, *yip4a yip4b* and *spk1-4,* total proteins were extracted from five-day-old seedlings grown on 1/2x MS agar medium. Twenty micrograms of MBP-RIC1-conjugated agarose beads were added to the protein extracts and incubated at 4°C for 2 h on a rocking table. The beads were washed four times in wash buffer (25 mM HEPES, pH 7.4, 1 mM EDTA, 5 mM $MgCl_2$, 1 mM dithiothreitol, and 0.5% (v/v) Triton X-100) at 4°C. GTP-bound ROP proteins that were associated with the MBP-RIC1 beads were boiled and used for analysis by western blotting with a ROP2- specific antibody. ROP2 polyclonal antibodies was generated against the peptide QFFIDHPGAVPITTNQG (Abicode). Prior to the pull-down assay, a fraction of total proteins was analyzed by immunoblot assay to determine total ROP2 (GDP bound and GTP bound, blot on the left). The amount of the GTP-bound active form of ROP2 (blot on the right) was normalized to that of total ROP2.

Development • Supplementary information

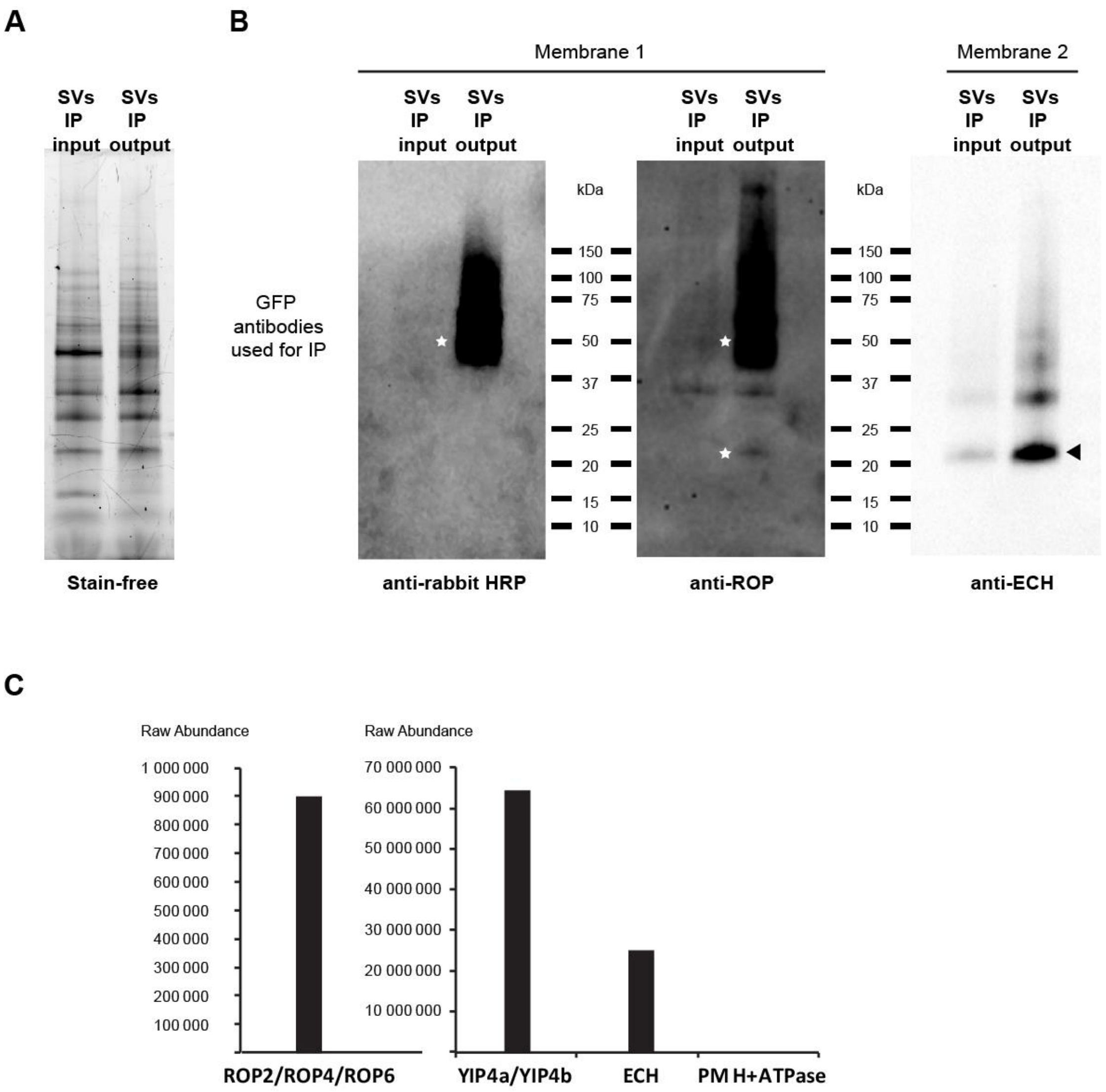

Fig. S7. ROP and YIP4 proteins are found in SYP61-positive vesicles.

(A) Stain-free loading control (Biorad) of SYP61 secretory vesicle (SVs) immunoprecipitation (IP) input from a sucrose-gradient with total membrane fraction and IP output from the bead fraction displaying equal loading. (B) Western blot of same loading as in (A) using first the goat anti-rabbit HRP coupled secondary antibody, only, without any primary antibody (blot on the left). On this blot we could clearly see the GFP antibodies around 50 kDa but could not detect any band around 20 kDa. The same membrane was then incubated with anti-ROP antibodies followed by incubation with the secondary antibody, which revealed a band around 21 kDa in the SYP61 IP fraction. Moreover, blotting anti-ECH antibody on another membrane (membrane 2) loaded exactly the same way as membrane 1 revealed a very strong enrichment of the targeted compartment SYP61 where ECH resides. (C) Label-free proteomics revealed the presence of ROP2, ROP4 and ROP6 common peptides (3 common peptides detected, hence it is impossible to differentiate the different ROPs), YIP4a and YIP4b (10 peptides detected), ECHIDNA (8 peptides detected) but not PM-H+-ATPase (AT2G24520) a marker for the plasma membrane, indicating no contamination from the PM fraction-in the SVs.

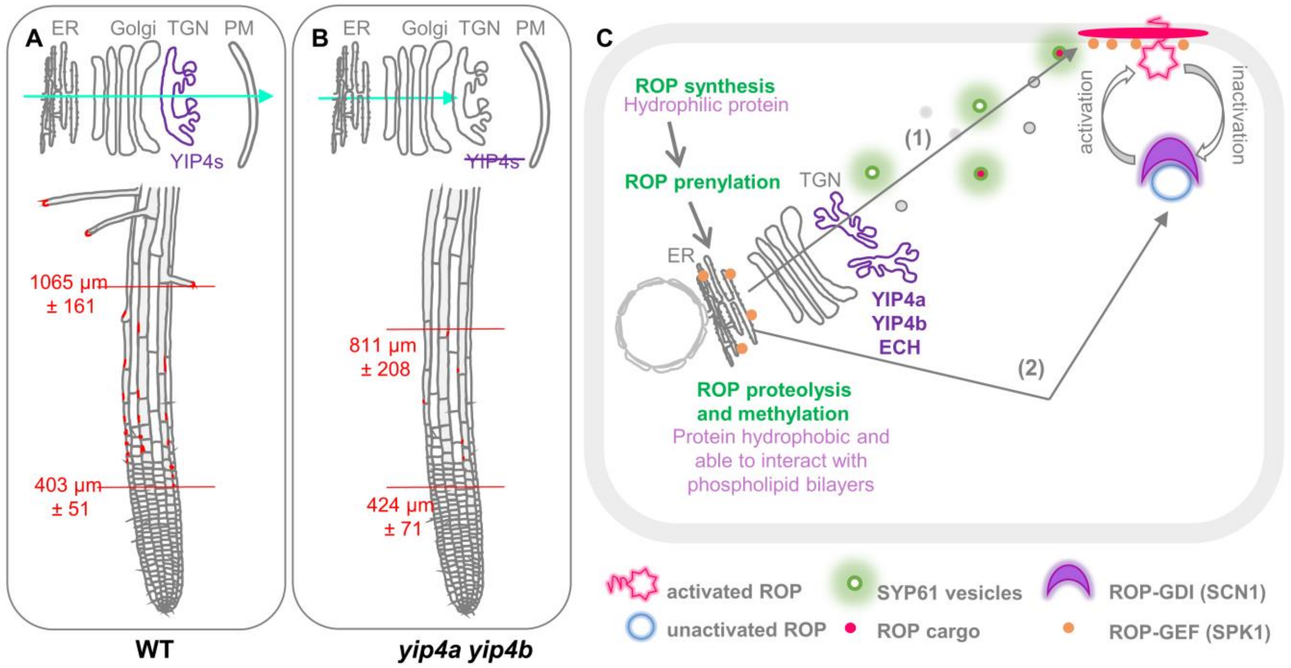

Fig.S8. Schematic model of the putative role of the secretory pathway in the plasma membrane-localization of type-I ROPs.

(A) Wild-type roots displaying a functional secretory system contain numerous ROP patches (red) at incipient hair initiation sites which persist at the tip of the growing hair. (B) When YIP4s (and their complex partner ECHIDNA) are lacking, the secretion of some cargos en route to the PM is impaired (Boutte et al., 2013; Gendre et al., 2013), likely due to a defect in secretory vesicle formation (Boutte et al., 2013). Mutants then lack hairs and show a drastic reduction in number and intensity of ROP patches. (C) Type-I ROP (ROP1-8) proteins synthesized at free polysomes in the cytosol require prenylation and the posttranslational modification of the C-termini at the surface of the endoplasmic reticulum (ER) for their membrane anchoring (Wright and Philips, 2006; Bracha-Drori et al., 2008; Feiguelman et al., 2018). Furthermore, the presence of ROP-GEF SPK1 both at the ER exit site (Zhang et al., 2010) and on punctae associated with the PM (Yanagisawa et al., 2018) reinforces the importance of the ER. Studies in yeast and mammals suggest that that Rho/Rac/Cdc42 could exit directly from the ER via a complex with RhoGDI after having been extracted from the organelle membrane (route 2, fast) or via vesicles (route 1, slow)(Slaughter et al., 2009; Garcia-Mata et al., 2011; Watson et al., 2014). By analogy, ROP may reach the PM by two different means in plants. ROPs are found in SYP61-vesicles, EYFP-ROP2 recovery after photobleaching is impaired at the plasma membrane and intensity of ROP patches is decreased in the secretion-defective *yip4a yip4b* mutant, suggesting that a part of ROP is taking the secretory pathway on its way to the PM. It remains open whether secretion of other factors crucial to

Development • Supplementary information

activate ROP at the PM, like ROP-GEF, are also impaired in *yip4a yip4b* resulting in the decrease in ROP patches. In addition, tight regulation of the ROP activation/inactivation cycle keeps ROP polarized. Data from yeast indicates that the initial polar aggregation of Cdc42 seems highly dependent on the GTPase cycles and the cytosolic pool of GTPase and only partially dependent of secretion via actin filaments (Wedlich-Soldner et al., 2004). Hence, a contribution by both the secretory pathway and the cytosolic ROP pool is feasible.

Supplemental references

Boutte, Y., Jonsson, K., McFarlane, H. E., Johnson, E., Gendre, D., Swarup, R., Friml, J., Samuels, L., Robert, S. and Bhalerao, R. P. (2013). ECHIDNA-mediated post-Golgi trafficking of auxin carriers for differential cell elongation. *Proc Natl Acad Sci U S A* **110**, 16259-64.

Bracha-Drori, K., Shichrur, K., Lubetzky, T. C. and Yalovsky, S. (2008). Functional analysis of Arabidopsis postprenylation CaaX processing enzymes and their function in subcellular protein targeting. *Plant Physiol* **148**, 119-31.

Feiguelman, G., Fu, Y. and Yalovsky, S. (2018). ROP GTPases Structure-Function and Signaling Pathways. *Plant Physiol* **176**, 57-79.

Garcia-Mata, R., Boulter, E. and Burridge, K. (2011). The 'invisible hand': regulation of RHO GTPases by RHOGDIs. *Nat Rev Mol Cell Biol* **12**, 493-504.

Gendre, D., McFarlane, H. E., Johnson, E., Mouille, G., Sjodin, A., Oh, J., Levesque-Tremblay, G., Watanabe, Y., Samuels, L. and Bhalerao, R. P. (2013). Trans-Golgi network localized ECHIDNA/Ypt interacting protein complex is required for the secretion of cell wall polysaccharides in Arabidopsis. *Plant Cell* **25**, 2633-46.

Kiefer, C. S., Claes, A. R., Nzayisenga, J. C., Pietra, S., Stanislas, T., Huser, A., Ikeda, Y. and Grebe, M. (2015). Arabidopsis AIP1-2 restricted by WER-mediated patterning modulates planar polarity. *Development* **142**, 151-61.

Slaughter, B. D., Smith, S. E. and Li, R. (2009). Symmetry breaking in the life cycle of the budding yeast. *Cold Spring Harb Perspect Biol* **1**, a003384.

Stanislas, T., Huser, A., Barbosa, I. C., Kiefer, C. S., Brackmann, K., Pietra, S., Gustavsson, A., Zourelidou, M., Schwechheimer, C. and Grebe, M. (2015). Arabidopsis D6PK is a lipid domain-dependent mediator of root epidermal planar polarity. *Nat Plants* **1**, 15162.

Watson, L. J., Rossi, G. and Brennwald, P. (2014). Quantitative analysis of membrane trafficking in regulation of Cdc42 polarity. *Traffic* **15**, 1330-43.

Wattelet-Boyer, V., Brocard, L., Jonsson, K., Esnay, N., Joubes, J., Domergue, F., Mongrand, S., Raikhel, N., Bhalerao, R. P., Moreau, P. et al. (2016). Enrichment of hydroxylated C24- and C26-acyl-chain sphingolipids mediates PIN2 apical sorting at trans-Golgi network subdomains. *Nat Commun* **7**, 12788.

Wedlich-Soldner, R., Wai, S. C., Schmidt, T. and Li, R. (2004). Robust cell polarity is a dynamic state established by coupling transport and GTPase signaling. *J Cell Biol* **166**, 889-900.

Wright, L. P. and Philips, M. R. (2006). Thematic review series: lipid posttranslational modifications. CAAX modification and membrane targeting of Ras. *J Lipid Res* **47**, 883-91.

Yanagisawa, M., Alonso, J. M. and Szymanski, D. B. (2018). Microtubule-Dependent Confinement of a Cell Signaling and Actin Polymerization Control Module Regulates Polarized Cell Growth. *Curr Biol* **28**, 2459-2466 e4.

Zhang, C., Kotchoni, S. O., Samuels, A. L. and Szymanski, D. B. (2010). SPIKE1 signals originate from and assemble specialized domains of the endoplasmic reticulum. *Curr Biol* **20**, 2144-9.
